# Patterns of Sustainability Capacity Among Organizations That Deliver the National Diabetes Prevention Program: A Latent Profile Analysis

**DOI:** 10.5888/pcd20.230067

**Published:** 2023-10-12

**Authors:** Lillian Madrigal, Regine Haardörfer, Michelle C. Kegler, Sarah Piper, Linelle M. Blais, Mary Beth Weber, Cam Escoffery

**Affiliations:** 1 Rollins School of Public Health, Emory University, Atlanta, Georgia

## Abstract

**Introduction:**

Since the launch of the National Diabetes Prevention Program (DPP) in 2010, more than 3,000 organizations have registered with the Centers for Disease and Control and Prevention to deliver the program; today, however, only approximately 2,000 organizations are registered, indicating challenges with sustainability. We used the Program Sustainability Assessment Tool (PSAT) to explore patterns of sustainability capacity among National DPP delivery organizations.

**Methods:**

We used data from a cross-sectional online survey conducted in August and September 2021 of staff members (N = 440) at National DPP delivery organizations. We conducted a latent profile analysis to identify latent subpopulations on the basis of respondent PSAT domain scores. Regression analyses were used to estimate associations between derived latent classes, PSAT scores, and respondent characteristics.

**Results:**

The 4-class model included 4 groups of capacity for program sustainability, ranging from low to high: low (class 1) with 8.0% of the sample, medium-low (class 2) with 22.0%, medium-high (class 3) with 41.6%, and high (class 4) with 28.4%. Program evaluation (mean score = 5.1 [SD = 1.4]) and adaptation (mean score = 5.3 [SD = 1.3]) were the domains with the highest scores, while funding stability (mean score = 4.0 [SD = 1.6]) and Partnerships (mean score = 4.0 [SD = 1.7]) had the lowest scores. In our sample of National DPP delivery organizations, most reported relatively high capacity for program sustainability, and key indicators associated with sustainability capacity were virtual delivery, location of delivery, funding sources, and organization type.

**Discussion:**

Similar to sustainability capacity findings from other PSAT studies, our study found that funding stability and partnerships are areas to strengthen. This insight is useful in sustainability planning at organizational and national levels across multiple programs.

**Summary**

**What is already known on this topic?**

The Program Sustainability Assessment Tool is used by health promotion programs to improve sustainability planning. Many organizations deliver the National Diabetes Prevention Program (DPP), but research on this program’s sustainability is limited.

**What is added by this report?**

National DPP respondents were classified into 4 groups by level of sustainability capacity and the key indicators associated with sustainability capacity: virtual delivery, location of delivery, funding sources, and organization type.

**What are the implications for public health practice?**

The National DPP and other chronic disease prevention programs can use our findings to support the use of the Program Sustainability Assessment Tool to assess capacity of National DPP delivery organizations and other programs and as a first step toward sustainability planning.

## Introduction

In 2020, an estimated 96 million adults in the US had prediabetes, a diagnosis that indicates a person is at risk for developing type 2 diabetes (1). For 20 years, the Diabetes Prevention Program (DPP), a lifestyle intervention to delay the onset of diabetes among people at high risk for diabetes, has been rigorously tested and adapted in multiple populations and formats (2,3). In 2010, the Centers for Disease Control and Prevention (CDC) launched the National DPP initiative to scale and sustain the intervention to make it widely available to the US population with prediabetes (4). CDC has invested in the program via multiple federal award mechanisms to support the infrastructure, implementation, and scaling of the program with the goal of these programs to become financially self-sustaining through various private and public payer models. Today, more than 2,000 National DPP organizations are registered to implement the year-long lifestyle-change program, a decrease from the more than 3,000 organizations that provided the National DPP from 2012 through 2019; this decrease indicates challenges in sustainability (5,6).

To make a population-level impact, evidence-based interventions need to be scaled *and sustained* (7). The field of implementation science defines sustainability as “the continued use of program components at sufficient intensity for the sustained achievement of desirable program goals and population outcomes” (7). The longer an intervention remains in place, the greater reach and effect it can have (8). Factors associated with sustainability include, among others, the adaptability of a program, support of champions and other key partners, program fit within an organization, perceived impacts and benefits of a program, and organization capacity (7,9,10).

Although people familiar with the National DPP know of many organizations that have been delivering the program for years, research is limited on the average length of delivery or sustainability in general. A 2019 study of 165 CDC-recognized organizations delivering the National DPP defined sustainability by using the RE-AIM (Reach, Effectiveness, Adoption, Implementation, and Maintenance) framework’s domain of maintenance (“the extent to which programs had potential for sustainability, measured by the number of delivery sites achieving full CDC recognition, the number of sites continuing to deliver the program without cooperative agreement funding, and organizational and financial support or program reimbursement from private or public payers”) (11). The study found that in 4 years (2012–2016), 132 sites (80%) had at least 12 months of participant data and 33 (25%) of these 132 achieved full CDC recognition. CDC recognition involves demonstrating how the delivery organization has effectively delivered the National DPP and met a set of CDC implementation and outcome standards. Because the National DPP takes 1 year to complete, 12 months of data is considered a short-term indicator of sustainability at best. No other sustainability findings, including factors predicting program sustainability, were reported.

A movement has begun in recent years to better define, operationalize, and measure sustainability of evidence-based public health programs (7,12,13). The Program Sustainability Assessment Tool (PSAT), developed in 2014 through a comprehensive review of tools measuring public health program sustainability, examines 8 domains that affect program sustainability capacity (14). The PSAT defines program sustainability capacity as the ability to maintain programming and its benefits over time. The PSAT has been used primarily as a planning tool at a single point in time and has not been tested in a predictive capacity (12). Understanding patterns in program sustainability capacity across organizations may be useful in understanding the landscape of National DPP delivery.

Latent profile analysis (LPA) is a statistical method that focuses on identifying subpopulations within a population based on a certain set of continuous variables into mutually exclusive groups or classes, called “latent profiles” (15,16). The term “latent” is used to describe the class membership that cannot be directly observed. At the organizational level, LPA has been used to examine patterns in contextual and organizational factors in community-based programs (17), clinician practices (18), and community readiness for programs (19) to better understand evidence-based program implementation and adoption. However, at the organizational level, to our knowledge, no studies have used LPA to examine program sustainability capacity among National DPP delivery organizations.

The objective of this study was to explore patterns of program sustainability capacity among organizations delivering the National DPP by using the Program Sustainability Assessment Tool (PSAT), to understand whether organizations can be categorized into distinct groups based on dimensions of their sustainability capacity and if those groups are associated with specific organizational characteristics. The findings from this research have the potential to support National DPP implementation by providing an understanding of sustainability capacity strengths and weaknesses across different organizations as well as recommendations for capacity building for sustainability at National DPP delivery organizations.

## Methods

This study used data from a cross-sectional online survey conducted in August and September 2021 of staff members at National DPP delivery organizations. The sample included staff members in the following key roles: lifestyle coaches, master trainers, and program coordinators. This study was reviewed and determined to be exempt by the Emory University Institutional Review Board (STUDY00002611).

### Measures

The survey instrument included 107 items: 23 items about the respondent and the respondent’s organization, 40 items from the Program Sustainability Assessment Tool (https://sustaintool.org/psat), and 38 Likert-scale items from the Consolidated Framework for Implementation Research (https://cfirguide.org/). Of the 23 items on respondent and organization characteristics, we analyzed 4 items of respondent characteristics (role at organization, gender, race and ethnicity, and age) and 12 items on organizational characteristics: 1) recognition in CDC’s Diabetes Prevention Recognition Program (DPRP); 2) length of program delivery, in years; 3) enrollment (the number of program participants enrolled to date in DPP); 4) the number of lifestyle coaches on staff; 5) the number of staff members who are not lifestyle coaches; 6) the number of staff members who are 100% dedicated to the National DPP; 7) type of organization; 8) size of organization; 9) program delivery mode (eg, in person, distance); 10) location (eg, rural, urban), 11) racial and ethnic composition of program participants; and 12) National DPP funding sources. Because of the large number of variables for respondent and organization characteristics and small sample sizes for some categories of race and ethnicity, we aggregated data to compare organizations that serve either White-only populations or non–White-only populations to understand differential reach to these audiences. This study focused on the analysis of the delivery organization characteristics and the PSAT items. We did not analyze data from the Consolidated Framework for Implementation Research.

The PSAT covers 8 sustainability domains: Environmental Support, Funding Stability, Partnerships, Organizational Capacity, Program Evaluation, Program Adaptation, Communications, and Strategic Planning. Items assessed the extent to which a program has or does the following on a Likert scale from 1 to 7 (1 = a little or no extent, 7 = to a very great extent). Domain scores are averaged and provide a PSAT score of 1 to 7, which indicates the level of sustainability capacity. The higher the score, the greater the sustainability capacity. The PSAT has been tested for psychometric properties in trainings and evaluations with more than 550 people and more than 250 unique programs at state and local levels (14,20). Reliability testing determined a high reliability, with Cronbach α for domain subscales ranging from 0.79 to 0.92 (14).

### Data collection and study sample

Study participants were recruited from the National DPP implementer population at Emory University’s Diabetes Training and Technical Assistance Center (DTTAC). In the past 10 years, DTTAC has directly trained more than 5,000 lifestyle coaches representing more than 2,000 organizations across all 50 states. The survey was distributed to an email contact list of 6,470 National DPP delivery organizations that have participated in Emory’s DTTAC programs or subscribed to the DTTAC newsletter. The first 336 respondents received a $15 Amazon gift card for their participation. After data cleaning for completion, we included 586 responses (9% response rate) in the analysis. Of those 586 respondents, 440 (75%) had a calculable PSAT score. According to PSAT instructions, averages are totaled and nonresponse items are excluded; in other words, we excluded respondents who did not respond to PSAT items. PSAT scores can be calculated by answering one or more of the items. Of those who responded to the PSAT questions, an average 35 (SD, 8.9) of 40 questions were answered, and response completion ranged from 1 to 40 items.

### Descriptive analysis

Data were exported from Qualtrics and analyzed by using SAS software version 9.4 (SAS Institute Inc). After data were cleaned, we ran standard descriptive statistics (eg, frequencies, distributions, means). We scaled (divided by 100) the enrollment-to-date variable to assist with comparisons across other variables after removing 4 outliers above the 99th percentile. We compared respondents with and without a PSAT score; we used χ^2^ and *t *tests to detect significant differences between groups. The level of significance was set at *P* <.05 for all analyses.

**PSAT item internal consistency** *.* We calculated the means and SDs for all 40 PSAT items along with domain averages and the total PSAT score average. We calculated Cronbach α for all domains and the total PSAT score, measuring the internal consistency of the items within each domain scale and the domains together to form the PSAT score.

**LPA.** LPA is a person-centered approach that classifies respondents into mutually exclusive profiles, groups, classes, or clusters (16). First, the researcher selects numerous groups or classes to assign the respondent to (ie, asks the statistical program to allocate respondents into 2 classes, 3 classes, 4 classes, and so on, by using the data available). After analyzing all respondent data for each PSAT domain score, the respondents are assigned a probability of being part of each derived class and assigned to a class according to their highest posterior probability. The assigned class is treated like an unobserved categorical variable, where its value indicates which profile a respondent belongs to with a certain degree of probability (16).

This LPA included the 8 PSAT domain score variables. We used Mplus version 8.3 (Muthén and Muthén) to run a succession of 8 models. Cases with missing items were estimated in Mplus by using full information maximum likelihood. The selection of the best model included reviewing the fit indices and information criteria; latent class proportions and sizes; and the researchers’ interpretation and entropy of the latent classes. All entropy values in the models run were greater than 0.85. After review of fit indices, class proportions, and the visual profiles of each, we selected the 4-class model as the best fit and most meaningful output of class proportions.

To estimate associations between derived latent classes and organization characteristics, we conducted multivariable multinomial logistic regression using class 1 (low sustainability score) as the reference group. Respondent organization characteristics in the multivariable multinomial logistic regression included the following variables: the number of program participants enrolled to date, staffing (number of lifestyle coaches on staff, number of staff dedicated 100% to National DDP), organization type, organization size, program delivery mode, location, programs with White only and non-White only populations enrolled, and National DPPP funding sources. The final sample for the multivariable multinomial logistic regressions included 259 respondents, which was smaller than our sample of 440 respondents to the PSAT questions because of data missing at random.

### Bivariate and multivariable regression

To gain a better understanding of relationships between variables and compare with the LPA results, in addition to the LPA, we conducted bivariate and multivariable regressions for all key organization characteristic variables with the PSAT score as the outcome. We calculated Pearson correlation coefficients for variables that captured similar dimensions (multicollinearity). For the full multivariable regression model, we combined variables (racial and ethnic composition of program participants and program delivery mode) or eliminated variables (recognition in CDC’s DPRP, length of program delivery, staff members who are not DPP lifestyle coaches) on the basis of degree of correlation and theoretical overlap with other variables. The final multivariable regression model included 21 variables for organization characteristics.

After reviewing patterns of missing data, we concluded that data were missing at random, that is, missingness was related only to variables that were collected. We used multiple imputations in SAS software version 9.4 to handle data missing at random for the enrollment (30% missing) and organization size (20% missing). Twenty-one variables on organization characteristics from our main analysis plus 4 auxiliary organization variables related to missingness (DPRP status, years of program delivery, non-lifestyle coach staff, and health care organization as reference group for organization type) were added to the imputation model. We used the fully conditional method to handle the continuous, ordinal, and dichotomous variables. We created a total of 10 imputed data sets and compared these results with the original regression model and the LPA multivariable multinomial logistic regression.

## Results

Of the 440 survey respondents with a PSAT score, 399 (90.7%) were lifestyle coaches, 387 (88.0%) were women, 264 (60.0%) were White, and 288 (65.4%) were aged 35 to 64 years (Table 1). Most (52.5%) respondents belonged to organizations with full recognition in CDC’s DPRP. The mean (SD) length of program delivery was 4.6 (3.1) years (median, 4; IQR, 3.0–6.0), and mean (SD) enrollment to date was 1,991 (28,325) program participants (Table 2). The most common types of respondent organizations were health care and hospitals (31.4% of respondents); community-based health care (eg, community health centers, federally qualified health centers, Indian Health Service; 23.6% of respondents); health insurers, employers, and other (eg, private businesses; 15.5% of respondents); and government agencies (13.4% of respondents).

**Table 1:** Characteristics of Respondents (N = 440) to Survey on Program Sustainability Capacity Among Staff at National DPP Delivery Organizations, August–September 2021^a^

Characteristic	No. (%)
**Role at organization (could choose >1)**	
Lifestyle coach	399 (90.7)
Program coordinator	182 (41.4)
Master trainer	48 (10.9)
**Respondent gender**	
Woman	387 (88.0)
Man	35 (8.0)
Other	2 (0.5)
Missing	16 (3.6)
**Respondent race and ethnicity (could choose >1)**	
American Indian or Alaska Native	22 (5.0)
Asian	14 (3.2)
Black or African American	75 (17.0)
Hawaiian Native or Pacific Islander	1 (0.2)
Hispanic/Latino	52 (11.8)
White or Caucasian	264 (60.0)
Other	3 (0.7)
Missing	20 (4.5)
**Respondent age, y**	
<25	13 (3.0)
25–34	86 (19.5)
35–44	97 (22.0)
45–54	92 (20.9)
55–64	99 (22.5)
≥65	36 (8.2)
Missing	17 (3.9)

Abbreviation: DPP, Diabetes Prevention Program.

a Of 586 respondents to the survey, 440 (75%) had a calculable score for the Program Sustainability Assessment Tool and were included in the analysis.

**Table 2:** Characteristics of Organizations as Reported by Respondents (N = 440) to Survey on Program Sustainability Capacity Among Staff at National DPP Delivery Organizations, August–September 2021^a^

Characteristic	No. (%) of respondents	Value
**Recognition in CDC’s Diabetes Prevention Recognition Program**		
Full recognition	231 (52.5)	—
Pending or preliminary	112 (25.5)	—
None	34 (7.7)	—
I do not know or missing	63 (14.3)	—
**Program statistics, mean (SD)/median (IQR)**		
Length of National DPP delivery	419 (95)	4.6 (3.1) [0–20]/4.0 (3–6)
Enrollment (program participants enrolled in National DPP to date)	313 (71)	1,991 (28,325)/60 (175)
Enrollment scaled^b^	309 (70)	2.0 (3.8)/4.0 (6.0)
**Staffing, mean (SD)/median (IQR)**		
Lifestyle coaches on staff	430 (97.7)	7.4 (13.5)/4 (1–8)
Staff members who are not DPP lifestyle coaches	424 (96.4)	2.1 (8.2)/0 (0–2)
Staff members 100% dedicated to National DPP	425 (96.6)	2.0 (8.7)/0 (0–1)
**Organization type**		
Health care or hospital	138 (31.4)	—
Community-based health care	104 (23.6)	—
Community-based organization	40 (9.1)	—
Government agency	59 (13.4)	—
Academic institution	30 (6.8)	—
Health insurer, employer, other	68 (15.5)	—
Missing	1 (0.2)	—
**Organization size (no. of people served annually across all programs)**		
Small (0–1,000)	139 (31.6)	—
Medium (1,000–50,000)	151 (34.3)	—
Large (>50,000)	55 (12.5)	—
I don’t know or missing	95 (21.6)	—
**Program delivery mode (could choose >1)**		
In-person small group (meetings with ≤20 participants)	219 (49.8)	—
In-person large group (meetings with ≥21 participants)	14 (3.2)	—
Distance (interacting live with all participants as a group using video and/or audio)	252 (57.3)	—
Online (using a platform for participants to engage with the content on their own — not a live group meeting)	76 (17.3)	—
Hybrid (combination of modes)	105 (23.9)	—
Virtual (distance, online, hybrid)	343 (78.0)	—
Other	27 (6.1)	—
**Location (could choose >1)**		
Rural	181 (41.1)	—
Suburban	137 (31.1)	—
Urban	182 (41.4)	—
**Racial and ethnic populations primarily enrolled in organization’s National DPP (could choose >1)**		
American Indian or Alaska Native	53 (12.0)	—
Asian	44 (10.0)	—
Black	213 (48.4)	—
Hawaiian Native or Pacific Islander	9 (2.0)	—
Hispanic/Latino	147 (33.4)	—
White	298 (67.7)	—
Other	21 (4.8)	—
Missing	7 (1.6)	—
**Comparison of programs with White only and non-White only populations enrolled**		
White only	106 (24.1)	—
Non-White only	135 (30.7)	—
**National DPP funding sources (could choose >1)**		
Grant funding	183 (41.6)	—
State or local government funding	103 (23.4)	—
Federal government or CDC	99 (22.5)	—
Medicare or Medicaid	66 (15.0)	—
State employee coverage benefits	24 (5.5)	—
Missing	96 (21.8)	—

Abbreviations: —, does not apply; CDC, Centers for Disease Control and Prevention; DPP, Diabetes Prevention Program.

a Of 586 respondents to the survey, 440 (75%) had a calculable score for the Program Sustainability Assessment Tool and were included in the analysis.

b Enrollment-to-date variable was scaled (divided by 100) to assist with comparisons across other variables after removing 4 outliers above the 99th percentile.

Most (78.0%) respondents reported offering programs in some type of virtual mode (distance, 57.3%; online, 17.3%; hybrid, 23.9%; no response, 1.5%). Respondents reported mostly enrolling White (67.7% of respondents), Black (48.4% of respondents), and Hispanic or Latino (33.4% of respondents) populations. Respondents indicated that their organization was primarily funded or supported by grants (41.6% of respondents), state or local government (23.4% of respondents), or the federal government or CDC (22.5% of respondents) (Table 2).

When we compared the 440 respondents with a PSAT score with the 146 respondents who did not answer the PSAT items, one significant difference was that respondents with a PSAT score were significantly more likely to be program coordinators (41.1% vs 27.4%; *P* =.003) for the National DPP (a role more heavily involved with the program at the organizational level). Almost all (93.8%; 137 of 146) respondents who did not complete the PSAT also did not answer the demographic questions, which were at the end of the survey, indicating they simply did not complete the survey.

### PSAT scores

Mean (SD) scores for individual items in the PSAT instrument ranged from 3.6 (1.9) to 5.6 (1.4) (Table 3). All items and domain means were slightly positively skewed. By domain, program evaluation (5.1 [1.4]) and adaptation (5.3 [1.3]) were rated highest, whereas funding stability (4.0 [1.6]) and partnerships (4.0 [1.7]) were rated lowest. The mean (SD) PSAT score was 4.6 (1.3).

**Table 3:** PSAT Item Frequencies and Mean Domain Scores for Respondents (N = 440) to Survey on Program Sustainability Capacity Among Staff at National DPP Delivery Organizations, August–September 2021^a^

PSAT domain and item	No. of respondents	Mean (SD)^b^
**Environmental Support**		
1. Champions exist who strongly support our program.	409	4.9 (1.7)
2. Our program has strong champions with the ability to garner resources.	409	4.5 (1.7)
3. Our program has leadership support from within the larger organization.	418	4.9 (1.6)
4. Our program has leadership support from outside of the organization.	384	4.3 (1.8)
5. Our program has strong public support.	397	4.2 (1.7)
Overall domain	428	4.6 (1.5)
**Funding Stability**		
1. Our program exists in a supportive state economic climate.	351	4.2 (1.6)
2. Our program implements policies to help ensure sustained funding.	348	4.1 (1.8)
3. Our program is funded through a variety of sources.	346	3.8 (2.0)
4. Our program has a combination of stable and flexible funding.	340	3.7 (1.9)
5. Our program has sustained funding.	357	3.8 (1.9)
Overall domain	386	4.0 (1.6)
**Partnerships**		
1. Diverse community organizations are invested in the success of our program.	377	3.9 (1.8)
2. Our program communicates with community leaders.	386	4.4 (1.8)
3. Community leaders are involved with our program.	374	3.8 (1.9)
4. Community members are passionately committed to our program.	379	4.0 (1.9)
5. The community is engaged in the development of our program goals	373	3.6 (1.9)
Overall domain	408	4.0 (1.7)
**Organizational Capacity**		
1. Our program is well integrated into the operations of the organization.	407	4.8 (1.7)
2. Organizational systems are in place to support the various program needs.	411	4.8 (1.7)
3. Leadership effectively articulates the vision of our program to external partners.	401	4.6 (1.8)
4. Leadership efficiently manages staff and other resources.	412	4.8 (1.7)
5. Our program has adequate staff to complete the program’s goals.	413	4.8 (1.8)
Overall domain	422	4.7 (1.5)
**Program Evaluation**		
1. Our program has the capacity for quality program evaluation.	405	5.3 (1.5)
2. Our program reports short-term and intermediate outcomes.	392	5.2 (1.5)
3. Evaluation results inform program planning and implementation.	389	5.1 (1.5)
4. Program evaluation results are used to demonstrate successes to funders and other key stakeholders.	363	5.0 (1.7)
5. Our program provides strong evidence to the public that the program works.	382	5.0 (1.6)
Overall domain	412	5.1 (1.4)
**Program Adaptation**		
1. Our program staff periodically reviews the evidence base.	393	5.0 (1.6)
2. Our program adapts strategies as needed.	404	5.4 (1.4)
3. Our program adapts to new science.	389	5.4 (1.4)
4. Our program proactively adapts to changes in the environment.	404	5.6 (1.4)
5. Our program makes decisions about which components are ineffective and should not continue.	381	5.2 (1.5)
Overall domain	409	5.3 (1.3)
**Communications**		
1. Our program has communication strategies to secure and maintain public support.	384	4.5 (1.7)
2. Our program staff communicate the need for the program to the public.	386	4.7 (1.7)
3. Our program is marketed in a way that generates interest.	395	4.6 (1.7)
4. Our program increases community awareness of the issue (prediabetes or diabetes).	394	5.0 (1.6)
5. Our program demonstrates its value to the public.	395	4.9 (1.7)
Overall domain	413	4.7 (1.6)
**Strategic Planning**		
1. Our program plans for future resource needs.	390	5.0 (1.6)
2. Our program has a long-term financial plan.	344	4.1 (1.8)
3. Our program has a sustainability plan.	358	4.3 (1.8)
4. Our program’s goals are understood by all stakeholders.	365	4.4 (1.7)
5. Our program clearly outlines role and responsibilities for all stakeholders.	359	4.4 (1.8)
Overall domain	395	4.5 (1.6)
**Overall PSAT score**	**440**	**4.6 (1.3)**

Abbreviations: —, does not apply; CDC, Centers for Disease Control and Prevention; DPP, Diabetes Prevention Program; PSAT, Program Sustainability Assessment Tool.

a Of 586 respondents to the survey, 440 (75%) had a calculable score for the Program Sustainability Assessment Tool and were used in the analysis.

b Scaled from 1 to 7; the higher the score, the greater the sustainability capacity.

### Latent profile analysis

The model consisted of 4 classes with mean domain scores that ranged from low to high: class 1 had low program sustainability (8.0% of the sample), class 2 had medium-low program sustainability (22.0% of the sample), class 3 had medium-high program sustainability (41.6% of the sample), and class 4 had high program sustainability (28.4% of the sample) (Figure).

**Figure:**
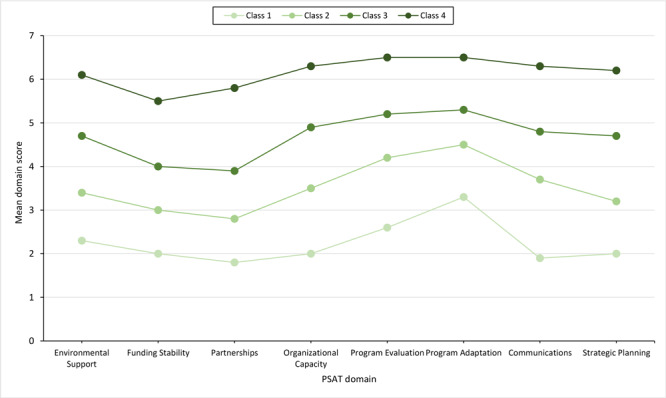
Four-class model of Program Sustainability Assessment Tool domains in a latent profile analysis of patterns of sustainability capacity among organizations that deliver the National Diabetes Prevention Program. Class 1 had low program sustainability (8.0% of the sample), class 2 had medium-low program sustainability (22.0% of the sample), class 3 had medium-high program sustainability (41.6% of the sample), and class 4 had high program sustainability (28.4% of the sample).

**Table:** 

Domain	Class 1: low program sustainability (8.0%, n = 35)	Class 2: medium-low program sustainability (22.0%, n = 97)	Class 3: medium-high program sustainability (41.6%, n = 183)	Class 4: high program sustainability (28.4%, n = 125)
Environmental Support	2.3	3.4	4.7	6.1
Funding Stability	2	3	4	5.5
Partnerships	1.8	2.8	3.9	5.8
Organizational Capacity	2	3.5	4.9	6.3
Program Evaluation	2.6	4.2	5.2	6.5
Program Adaptation	3.3	4.5	5.3	6.5
Communications	1.9	3.7	4.8	6.3
Strategic Planning	2	3.2	4.7	6.2

That we found no distinct patterns among classes indicates that all 8 PSAT domains were consistently scored across capacity levels, strengthening the evidence for internal consistency of the PSAT score, which is the average of all 8 domain scores. All organizations, despite capacity level, tended to have the same areas of strength and weakness. Program evaluation and adaptation had the highest scores in all classes across the model, whereas funding stability and partnerships had the lowest scores. This pattern matches the pattern of domain averages in the raw PSAT scores (Table 2), further supporting internal consistency of the PSAT score.

### Multivariable multinomial logistic regression

In the regression analyses of data from 259 survey respondents, compared with the class with low program sustainability (class 1), all other classes had on average 5.68 times (95% CI, 1.21–27.07) greater likelihood of having obtained grant funding to support their National DPP (Table 4). Compared with class 1, the class with medium-to-low program sustainability (class 2) was 4.36 times (95% CI, 1.08–17.67) more likely to be supported by state or local government funding, while the class with medium-high program sustainability (class 3) was less likely to have state employee coverage benefits for the National DPP (0.05, 95% CI, 0.003–0.84). Lastly, the class with high program sustainability (class 4) was 7.99 times (95% CI, 1.07–59.70) more likely than class 1 to be a government agency or academic institution.

**Table 4:** Four-Class Model Multivariable Multinomial Logistic Regression: Associations Between Organization Characteristics and Latent Profiles From Survey on Program Sustainability Capacity Among Staff at National DPP Delivery Organizations, August–September 2021^a^

Variable	Estimate (95% CI) [*P* value]^b^		
	Class 2: medium-low sustainability scores (22.0% of respondents)	Class 3: medium-high sustainability scores (41.6% of respondents)	Class 4: high sustainability scores (28.4% of respondents)
Enrollment (no. of program participants enrolled in National DPP to date)	1.33 (0.80–2.23) [.28]	1.27 (0.76–2.12) [.36]	1.44 (0.86–2.40) [.16]
**Staffing**			
No. of lifestyle coaches on staff	1.12 (0.89–1.42) [.34]	1.14 (0.90–1.43) [.28]	1.15 (0.91–1.45) [.24]
No. of staff 100% dedicated to National DPP	0.88 (0.44–1.76) [.71]	1.30 (0.72–2.37) [.38]	1.38 (0.75–2.51) [.30]
**Organization type**			
Community-based health care	0.76 (0.19–3.09) [.70]	1.68 (0.46–6.16) [.43]	0.92 (0.22–3.90) [.91]
Community-based organization	0.47 (0.06–3.50) [.46]	1.04 (0.16–6.83) [.97]	1.15 (0.16–8.12) [.89]
Government agency or academic	2.21 (0.29–17.00) [.45]	5.46 (0.75–39.91) [.09]	7.99 (1.07–59.70) [.04]
Health insurer, employer, other	1.72 (0.31–9.53) [.54]	2.56 (0.48–13.61) [.27]	1.62 (0.28–9.33) [.59]
**Organization size (no. of people served annually across all programs)**			
Medium vs small	1.01 (0.31–3.28) [.98]	1.62 (0.52–5.05) [.40]	1.11 (0.34–3.68) [.86]
Large vs small	0.31 (0.05–1.81) [.19]	0.66 (0.12–3.59) [.63]	0.55 (0.10–3.05) [.49]
**Program delivery model**			
In-person (small or large group)	2.44 (0.81–7.37) [.11]	1.76 (0.62–5.05) [.29]	1.38 (0.46–4.18) [.57]
Virtual (distance, online, hybrid)	0.97 (0.29–3.20) [.95]	1.20 (0.37–3.85) [.76]	1.53 (0.42–5.51) [.52]
**Location**			
Rural	1.40 (0.15–12.89) [.77]	0.57 (0.06–5.16) [.62]	0.35 (0.04–3.27) [.35]
Suburban	2.77 (0.31–24.68) [.36]	2.42 (0.28–21.12) [.42]	1.77 (0.20–15.92) [.61]
Urban	3.44 (0.34–34.83) [.30]	1.80 (0.18–17.62) [.62]	1.73 (0.17–17.36) [.64]
**Programs with White only or non-White only populations enrolled**			
White only	2.54 (0.71–9.15) [.15]	1.96 (0.57–6.80) [.29]	1.31 (0.35–4.93) [.69]
Non-White only	2.35 (0.60–9.24) [.22]	1.80 (0.49–6.53) [.37]	1.90 (0.49–7.39) [.35]
**National DPP funding sources**			
Grant funding	7.06 (1.84–27.07) [.004]	5.30 (1.45–19.45) [.01]	4.66 (1.21–18.03) [.03]
Federal government or CDC	2.66 (0.50–14.29) [.25]	3.97 (0.82–19.20) [.09]	3.25 (0.62–16.95) [.16]
State or local government funding	4.36 (1.08–17.67) [.04]	2.99 (0.76–11.71) [.12]	0.89 (0.20–3.99) [.88]
State employee coverage benefits	0.43 (0.04–4.54) [.48]	0.05 (0.003–0.84) [.04]	0.34 (0.03–3.39) [.36]
Medicare or Medicaid	0.69 (0.13–3.76) [.67]	1.20 (0.25–5.77) [.82]	0.75 (0.14–4.08) [.74]

Abbreviations: CDC, Centers for Disease Control and Prevention; DPP, Diabetes Prevention Program.

a Final sample included 259 respondents, smaller than our sample of 440 respondents to the Program Sustainability Assessment Tool questions because of data missing at random.

b Reference group is class 1, low program sustainability scores (8.0% of respondents).

### Multivariable regression analysis

Since our LPA model demonstrated the internal consistency strength of the PSAT score, we also ran a multivariable regression model using PSAT score as the outcome with the multiple imputations data set to compare organizational characteristic predictors. In multivariable regression analysis, the virtual delivery mode (0.49 PSAT points; 95% CI, 0.19–0.79;* t* = 3.21; *P* =.001) and rural location (−0.48 PSAT points; 95% CI, −0.80 to −0.16; *t* = −2.92; *P* =.004) were significantly associated with PSAT score (Table 5).

**Table 5:** Organizational Characteristics Associated With PSAT Scores in Survey on Program Sustainability Capacity Among Staff (N = 440) at National DPP Delivery Organizations, August–September 2021

Variable	Estimate^a^ (SE) [95%CI]	*P* value	Relative increase in variance, %	Fraction missing information
Intercept	4.23 (0.30) [−3.64 to 4.83]	<.001	9	0.08
Enrollment (no. of program participants enrolled in National DPP to date)	0.03 (0.02) [−0.02 to 0.07]	.25	13	0.12
**Staffing**				
No. of lifestyle coaches on staff	0 (0.01) [−0.01 to 0.01]	.64	6	0.06
No. of staff members 100% dedicated to National DPP	0.01 (0.01) [0 to 0.03]	.13	8	0.07
**Organization type**				
Community-based health care	0.11 (0.18) [−0.24 to 0.46]	.53	0	0
Community-based organization	0.38 (0.23) [−0.08 to 0.84]	.10	0	0
Government agency	0.12 (0.21) [−0.29 to 0.54]	.55	0	0
Academic	0.02 (0.26) [−0.49 to 0.54]	.93	1	0.01
Health insurer, employer, other	0.09 (0.19) [−0.28 to 0.47]	.62	0	0
**Organization size (no. of people served annually across all programs)**	0.01 (0.10) [−0.18 to 0.20]	.94	20	0.17
**Program delivery mode**				
In-person (small or large group)	−0.01 (0.13) [−0.26 to 0.23]	.91	0	0
Virtual (distance, online, hybrid)	0.49 (0.15) [0.19 to 0.79]	.001	1	0.01
**Location**				
Rural	−0.48 (0.16) [−0.80 to −0.16]	.004	1	0.01
Suburban	−0.12 (0.16) [−0.44 to 0.20]	.46	1	0.01
Urban	−0.17 (0.16) [−0.49 to 0.16]	.31	1	0.01
**Programs with White only or non-White only populations enrolled**				
White only	−0.13 (0.16) [−0.44 to 0.17]	.39	0	0
Non-White only	0.15 (0.15) [−0.15 to 0.45]	.32	0	0
**National DPP funding sources**				
Grant funding	0.20 (0.13) [−0.05 to 0.45]	.12	1	0.01
State or local government funding	0.26 (0.15) [−0.04 to 0.56]	.09	1	0.01
Federal government or CDC	−0.11 (0.15) [−0.40 to 0.19]	.49	0	0
Medicare or Medicaid	−0.14 (0.27) [−0.68 to 0.40]	.61	1	0.01
State employee coverage benefits	−0.05 (0.18) [−0.40 to 0.30]	.78	1	0.01

Abbreviations: CDC, Centers for Disease Control and Prevention; DPP, Diabetes Prevention Program; PSAT, Program Sustainability Assessment Tool.

a Estimates from 10 imputations.

## Discussion

The PSAT has been used by numerous health promotion programs to improve sustainability planning (21–26). This is the first study to use the PSAT to examine sustainability capacity in a sample of National DPP delivery organizations. The LPA did not identify any distinct patterns among PSAT respondents. Instead, the LPA categorized National DPP delivery organizations into a series of 4 classes that ranged from low PSAT scores to high PSAT scores. Overall, National DPP delivery organizations reported relatively high program sustainability capacity: most (70.0%) organizations were placed in the high (28.4%) or medium-high (41.6%) program sustainability class. These results provide evidence to support the reliability of the PSAT score to identify sustainability capacity.

In our study, similar to other studies using the PSAT, the funding stability domain had the lowest average score, and program adaptation and evaluation had the highest scores (14,25,27). In the multivariable multinomial logistic regressions of the 4-class LPA model, we also found that, compared with the low program sustainability class (class 1), organizations in the higher program sustainability classes (classes 2, 3, and 4) were more likely to have grant funding support for their National DPP efforts. Funding stability is often thought of as one of the most important factors in many sustainability frameworks and can influence other sustainability domains (10,28).

Although our study found external grant funding to be associated with sustainability, the National DPP has focused on reimbursements by insurance providers (employers, Medicare, Medicaid) (6), not on program implementation funding for financial sustainability. Only 15% of our sample indicated having Medicare and Medicaid support for their programs. Nationally, Medicare and Medicaid funding has posed challenges, namely that reimbursement rates are lower than actual organizational costs incurred, which may explain why Medicare and Medicaid were not significantly associated with sustainability capacity in our study (6,29). Future research could examine the extent to which organizations receive various forms of financial support, the duration of funding, and the impact of funding on sustainability outcomes for DPPs and other chronic disease programs.

Program evaluation and adaptation domains had the highest average scores in our sample, which may be explained by the robust standards and guidance provided by CDC’s DPRP. Many delivery organizations are familiar with collecting and analyzing their program data to submit to CDC to maintain their recognition status. Likewise, CDC has promoted various program adaptations to ensure the lifestyle change program curriculum is effective in the communities and populations where they are implemented. The National DPP’s Customer Service Center also provides technical assistance resources for tailoring program elements for various situations.

The LPA classes with higher program sustainability capacity (classes 3 and 4) rated the organizational capacity and communications domains highly. Findings were similar in other sustainability frameworks and studies (28). The organizational capacity domain refers to having the internal support and resources needed to effectively manage a program, while the communications domain focuses on strategic contact with interested parties and the public about the program. It is not surprising that organizations with resources, capacity, and strong communications can maintain their health promotion programming. Other factors related to these domains are characteristics such as effective leadership and support from champions (30), which in the PSAT are captured by the environment support domain, which was also highly rated by the groups in our sample with higher program sustainability capacity. Our previous research using the Consolidated Framework for Implementation Research to examine National DPP implementation also found that organizational capacity in terms of structural characteristics and leadership engagement affected the implementation outcome of reach (31).

One way in which organizational capacity strengths can be seen in our sample is through technology and staffing for virtual delivery. Before the COVID-19 pandemic, only a small number (121 in 2019) of organizations were offering the DPP program virtually (4); today, on the basis of our study findings and the technical assistance provided to delivery organizations, it is clear that many more organizations use virtual delivery methods out of necessity. In our study, 447 of 586 (76.3%) organizations reported virtual delivery. Benefits of virtual delivery include decreasing the cost of delivery; eliminating some logistical challenges, such as finding physical space for the program; and overcoming barriers to participant attendance, such as transportation or scheduling, while maintaining program outcomes on par with the in-person DPP (32). Using the PSAT score as the regression outcome, we found that organizations with any type of virtual delivery mode (online, distance, hybrid) were associated with higher sustainability capacity. In addition, virtual delivery allows for greater geographical reach of participants. In our analysis, the programs in rural locations were significantly associated with lower sustainability capacity scores. One implication of these data is that the National DPP should prioritize improving implementation in rural communities and virtual delivery modes to enhance their adoption and sustainability efforts.

Overall, organization type did not appear to influence sustainability capacity. In our multivariable multinomial logistic regressions of the 4-class LPA model, government and academic organizations were positively associated with the highest capacity class (class 4), which may indicate some benefit of being a government agency or academic institution when considering the long-term delivery and maintenance of the National DPP. Perhaps the staff may have more opportunities and capabilities (ie, grant writing skills) to receive funding at these types of institutions. Government agencies, such as health departments, also receive chronic disease funding and technical assistance, which may also affect their sustainability capacity. However, the lack of significant relationships between organization types and sustainability capacity supports CDC’s vision that a wide variety of organizations can deliver the National DPP. More research with a larger sample of National DPP organizations should be used to examine sustainability outcomes (eg, length of delivery, enrollment growth, maintained participant outcomes, financial self-sustainment) based on organization type.

The National DPP and other chronic disease prevention programs can use the findings of this study to support using the PSAT to assess sustainability capacity of their programs and as a first step toward sustainability planning. Future research can build on this work by using the PSAT to assess sustainability capacity at the point of program adoption and how well the measure can predict long-term sustainability outcomes (eg, length of delivery, sustained health outcomes, program growth). Providers of implementation technical assistance can also use the PSAT as the first step in an intervention to build sustainability capacity to help organizations with low PSAT scores. People providing technical assistance should pay particular attention to low levels of funding stability and help organizations secure new funding before current sources expire. Lastly, our study found that the National DPP program coordinator was more likely than respondents in other staff roles to complete the PSAT assessment. This finding indicates that the staff in the program coordinator role may be most able to complete organization-level assessments and should be engaged in sustainability planning and these types of studies in the future.

### Strengths and limitations

Strengths of this study include the use of the PSAT, a reliable measure for sustainability capacity with a large and diverse sample of National DPP delivery organizations. Although we had problems with missing data, we used multiple imputation methods to account for these data and draw meaningful conclusions. Another strength includes the large number of organizational characteristic variables used to study associations with program sustainability capacity scores. One limitation is that we recruited our sample from Emory’s DTTAC contact list; differences between this group and the larger National DPP population of implementers may exist because of resources the Emory group may have received from Emory University. We were also unable to accurately assess whether multiple people from the same organization completed the survey because of inconsistencies in how respondents provided organization names. We acknowledge that this limitation may have resulted in cluster effects. However, upon review of the organization names provided, more than 70% of the data included 1 respondent per organization listed. This large proportion of singletons in the data indicates that clustering cannot be adequately accounted for through methods such as a random effects model because of biases in the random effects estimation (33). An opportunity for future research may be to examine differences in sustainability capacity perception between groups of people with different organizational roles (eg, leadership compared with implementation staff) and how to engage various people in sustainability planning.

The incomplete information on organization name highlights another limitation: the inability to use data directly from the CDC DPRP database due to the federal government’s data use restrictions. Because of these restrictions, we relied on respondents to self-report their organizational information, which was not always complete, even after our attempts to follow up with respondents with missing data. For future studies, we hope researchers will have increased access to CDC DPRP data to better evaluate these implementation science questions at the national level.

### Conclusion

We used the PSAT to describe the sustainability capacity of National DPP delivery organizations. Understanding sustainability capacity is important to help program implementers with program planning, delivery, and scaling. Maintaining a program over time allows for growth and evolution within an organization in ways such as increasing program offerings, participant enrollment, and reach to new populations (10). Increased delivery allows for more program impact locally, while understanding sustainability across organizations offers collective learning and best practices to scale-up and scale-out programs in different settings, organizations, and populations. With the limited resources we have in public health promotion, we must strive to understand levers, and develop plans and interventions to scale and sustain effective evidence-based programs as best we can.
